# Creating “wizards” on the library’s website

**DOI:** 10.5195/jmla.2018.511

**Published:** 2018-10-01

**Authors:** Berika Williams, Debra Berlanstein

**Affiliations:** Emerging Technologies and Web Librarian, Hirsh Health Sciences Library, Tufts University, 145 Harrison Avenue, Boston MA 02111; Associate Director, Hirsh Health Sciences Library, Tufts University, Boston, MA

## Abstract

**Objectives:**

The library website is the virtual front door to the variety of services that the authors’ library offers. Library staff found confusion arose focused on two areas: how to reserve rooms in the library and how to reuse images using best practices. Two interactive forms were created that would hide and reveal content based on choices and lead the patron to answers.

**Methods:**

Brainstorming meetings were held to create a flow chart that identified the specific questions to be answered that would lead users to a logical answer. Once the correct flow was created, we approached each of these challenges by creating a custom module decision tree, using code through Drupal’s application programming interface (API) for forms.

**Results:**

The image reuse decision tree went live on the library website on February 17, 2017, and the room reservation wizard went live on August 27, 2017. By the end of the spring 2018 semester on May 18, 2018, the room reservation wizard had been accessed 1,945 times and risen to number 7 on the list of top-accessed website pages, and the image reuse wizard had been accessed 484 times.

**Conclusions:**

The popularity of both the decision tree pages is exciting. Both “wizards” have empowered users to find an answer to their questions virtually, especially during nonbusiness hours.

The Hirsh Health Sciences Library serves the health sciences campus of Tufts University, which includes programs for the School of Dental Medicine, School of Medicine, Friedman School of Nutrition Science and Policy, Sackler School of Graduate Biomedical Sciences, Public Health program, Physician Assistant program, Jean Mayer US Department of Agriculture (USDA) Human Nutrition Research Center on Aging, and Tufts Medical Center.

The library website is the virtual front door to the variety of services that are offered. Based on the number of emails, reference questions, and inquiries that staff were receiving, we discovered that users of the website were confused about two areas: how to reuse, attribute, and employ fair use of images; and how to reserve rooms in the library. Many of these inquiries were time intensive to answer, were time sensitive, or were asked outside of our normal business hours. To address this need, we decided to create two “wizards,” which are interactive forms that provide choices, and hide and reveal content, based on users’ answers. These forms allow relevant user information to be collected and provide customized answers.

The need for the image reuse wizard came during a period where the library was fielding numerous questions on image reuse during the medical school’s transition to a new electronic syllabus platform. We needed a place to document this information but were not sure how to present it. An initial planning team was convened and collectively came up with the idea to use the functionality of our Drupal website to create a decision tree. Brainstorming meetings were held over a period of a semester to create and refine a flow chart that identified the specific questions to be answered that would lead users to a logical answer. Once we created the appropriate flow, we approached each of these challenges by creating a custom module decision tree, using code through Drupal’s application programming interface (API) for forms.

Based on this experience, a similar wizard was developed for room reservations to help mitigate the time it takes for staff to answer frequently asked questions about how to reserve rooms. This planning process, which involved creating a flow chart based on the questions that the Hirsh Library was receiving from users, was completed over a period of a few weeks.

Each wizard is an interactive form, hiding and revealing content based on the options that the user chooses. For example, the room reservation wizard begins with a question asking who users are—student, faculty, or staff—and moves forward based on their responses. The image reuse wizard begins by asking if users already have an image to use or wish to find one online. By answering the series of questions, users are led to an actual answer. If a more individualized answer is required, a contact form is provided.

The wizards are custom interactive forms using Drupal’s API for forms that allow more flexibility as to when to “hide” or “show” a question based on end users’ previous responses. It is also feasible to create a simple interactive form (with no development needed) by using Drupal’s user interface and adding one of the contributed modules, WebForms or Entityforms, to the user interface, then configuring the form to behave like staff want it to.

The image reuse decision tree went live on the library website on February 17, 2017, and the room reservation wizard went live on August 27, 2017. By the end of the spring 2018 semester, the room reservation wizard had been accessed 1,945 times and risen to number 7 on the list of top-accessed website pages, and the image reuse wizard had been accessed 484 times. We consider the use of the wizards a success, based on the high number of times that people are using these features and the reduction of reference questions and inquiries that we are receiving on these topics. Both decision trees have empowered users to find an answer to their questions, especially during nonbusiness hours.

## LINKS

- Room Reservation Wizard
- Image Reuse Wizard

